# The Emerging Global Threat of Salt Contamination of Water Supplies in Tidal Rivers

**DOI:** 10.1021/acs.estlett.5c00505

**Published:** 2025-07-02

**Authors:** Ming Li, Raymond G. Najjar, Sujay Kaushal, Alfonso Mejia, Robert J. Chant, David K. Ralston, Hans Burchard, Antonia Hadjimichael, Allison Lassiter, Xiaohong Wang

**Affiliations:** † Horn Point Laboratory, University of Maryland Center for Environmental Science, Cambridge, Maryland 21613, United States; ‡ Department of Meteorology and Atmospheric Science, 8082 The Pennsylvania State University, University Park, Pennsylvania 16802, United States; § Department of Geology, University of Maryland, College Park, Maryland 20740, United States; ∥ Department of Civil and Environmental Engineering, 8082 The Pennsylvania State University, University Park, Pennsylvania 16802, United States; ⊥ Department of Marine and Coastal Sciences, 242612 Rutgers University, New Brunswick, New Jersey 08901, United States; # Applied Ocean Physics and Engineering Department, 10627 Woods Hole Oceanographic Institution, Woods Hole, Massachusetts 02543, United States; 7 Department of Physical Oceanography, 545827 Leibniz Institute for Baltic Sea Research, Rostock, D-18055, Germany; 8 Department of Geosciences, 8082 The Pennsylvania State University, University Park, Pennsylvania 16802, United States; 9 Earth and Environmental Systems Institute (EESI), 8082 The Pennsylvania State University, University Park, Pennsylvania 16802, United States; 10 Department of City and Regional Planning, Weitzman School of Design, 6572 University of Pennsylvania, Philadelphia, Pennsylvania 19104, United States; 11 Department of Computer Science, 14726 Salisbury University, Salisbury, Maryland 21801, United States

**Keywords:** Tidal rivers, saltwater intrusion, freshwater salinization, water supplies, climate change

## Abstract

Salt contamination of water supplies in tidal rivers is a global problem, but it has received little attention beyond site-specific studies. Drought, sea-level rise, navigation channel dredging, and watershed land-use change increase the risk of salinization and threaten drinking water supplies, agricultural irrigation, and infrastructure (via corrosion). The emerging issue of salt contamination of water supplies in tidal rivers and its diverse impacts highlight the critical need for interdisciplinary research that must integrate knowledge from oceanography, hydrology, and water resource management. Here we elucidate oceanic and hydrological processes regulating saltwater intrusion into estuaries and tidal rivers as well as watershed processes driving enhanced chemical weathering and export of watershed salts into rivers. By synthesizing studies around the world, we discuss how sea-level rise, prolonged drought, and increasingly extreme weather events in a changing climate are driving more frequent saltwater intrusion events that threaten water security globally. We propose a convergent research agenda toward the development of a decision support tool for salinity management. Specifically we recommend making ion-specific measurements and developing hydrological–hydrodynamic models to simulate the transport of major salt ions. These models can then be combined with artificial intelligence algorithms and enhanced monitoring to explore management strategies with stakeholders.

## Graphical abstract

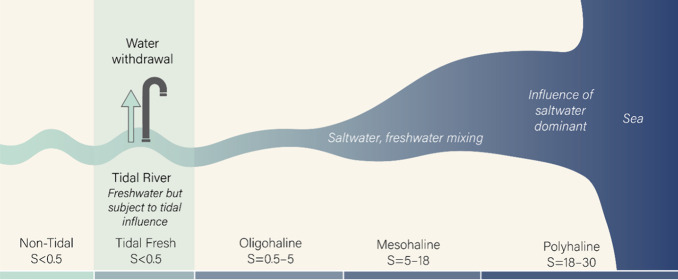

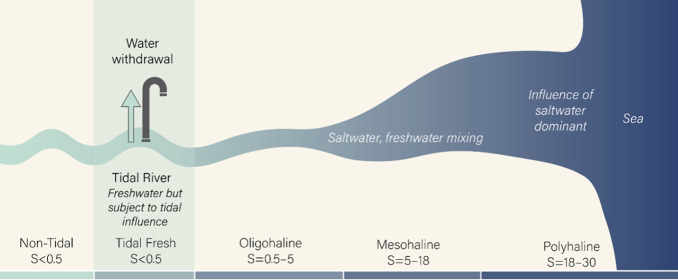

## 1 The Emerging Global Issue

About two-thirds of the global drinking water supply comes from surface waters, including tidal rivers. The World Health Organization recommends that drinking water should not contain more than 250 mg L^–1^ of chloride, and that high sodium levels (>20 mg L^–1^) in drinking water are linked to hypertensive disorders and developmental delays in children.^1–4^ Since seawater contains about 19,400 mg L^–1^ of chloride and 10,670 mg L^–1^ of sodium, saltwater intrusion poses a major threat to public health. Salt contamination of drinking water intakes in tidal rivers has made headlines worldwide in recent years. For example, the United States (US) Army Corps of Engineers had to barge freshwater to water treatment facilities in New Orleans to decrease the salinity to levels safe for drinking in fall 2023.^5^ A temporary emergency barrier was placed on the West False River in the Sacramento–San Joaquin Delta in June 2021 to slow saltwater intrusion from the ocean.^6^ Salt contamination of drinking water also occurred in the Chao Phraya River in 2020, where residents in Bangkok, Thailand, were urged to conserve water.^7^ The 2022 summer drought in Europe led to record low flows in the Rhine River and triggered emergency water conservation measures in The Netherlands.^8^ These events expose a void in understanding the salt contamination of water supplies in tidal rivers.

Saltwater intrusion is a global problem affecting many countries^9^ (Figure 1a). Several rivers in Africa are affected, including the Pungue River between Zimbabwe and Mozambique and the Incomati River in southeast Africa.^10^ In Europe, saltwater intrusion concerns range from the Mediterranean to the Atlantic and North Sea coasts, including the Po River Delta in Italy,^11^ the Garonne, Loire and Seine Rivers in France,^12^ the Rhine River in The Netherlands, and the Elbe, Weser and Ems estuaries in Germany.^13^ Many of Asia’s megacities are vulnerable to salt contamination of water supplies, including Shanghai on the Changjiang River,^14^ Zhuhai and Zhongshan on the Pearl River^15^ and several cities on the Ganges-Brahmaputra-Meghna Delta.^16^ In South America, saltwater intrusion affects the Valdivia River in Chile,^17^ the São Francisco River in Brazil and the Magdalena River in Colombia.^18^ In North America, saltwater intrusion affects rivers that drain into all three coasts, including the Delaware River (Figure S1 in the Supporting Information), the Hudson River (Figure S2),^19^ Sacramento–San Joaquin Delta–San Francisco Bay,^6^ the Mississippi River, and the Papaloapan River. Saltwater intrusion into tidal rivers is occurring not only in semiarid and Mediterranean-type climate regions, which are exposed to annually recurrent drought periods,^20,21^ but also in precipitation-rich temperate climates that may experience flash droughts.^22–24^ Equally serious to the problem of oceanic saltwater intrusion is freshwater salinization, the rise in salinity in the “fresh” end member of tidal rivers, owing to various anthropogenic activities within watersheds.^25,26^ Despite widespread reports of drinking water supplies being threatened by saltwater contamination, there is no global synthesis of the commonalities faced by these coastal systems.

**Figure 1:**
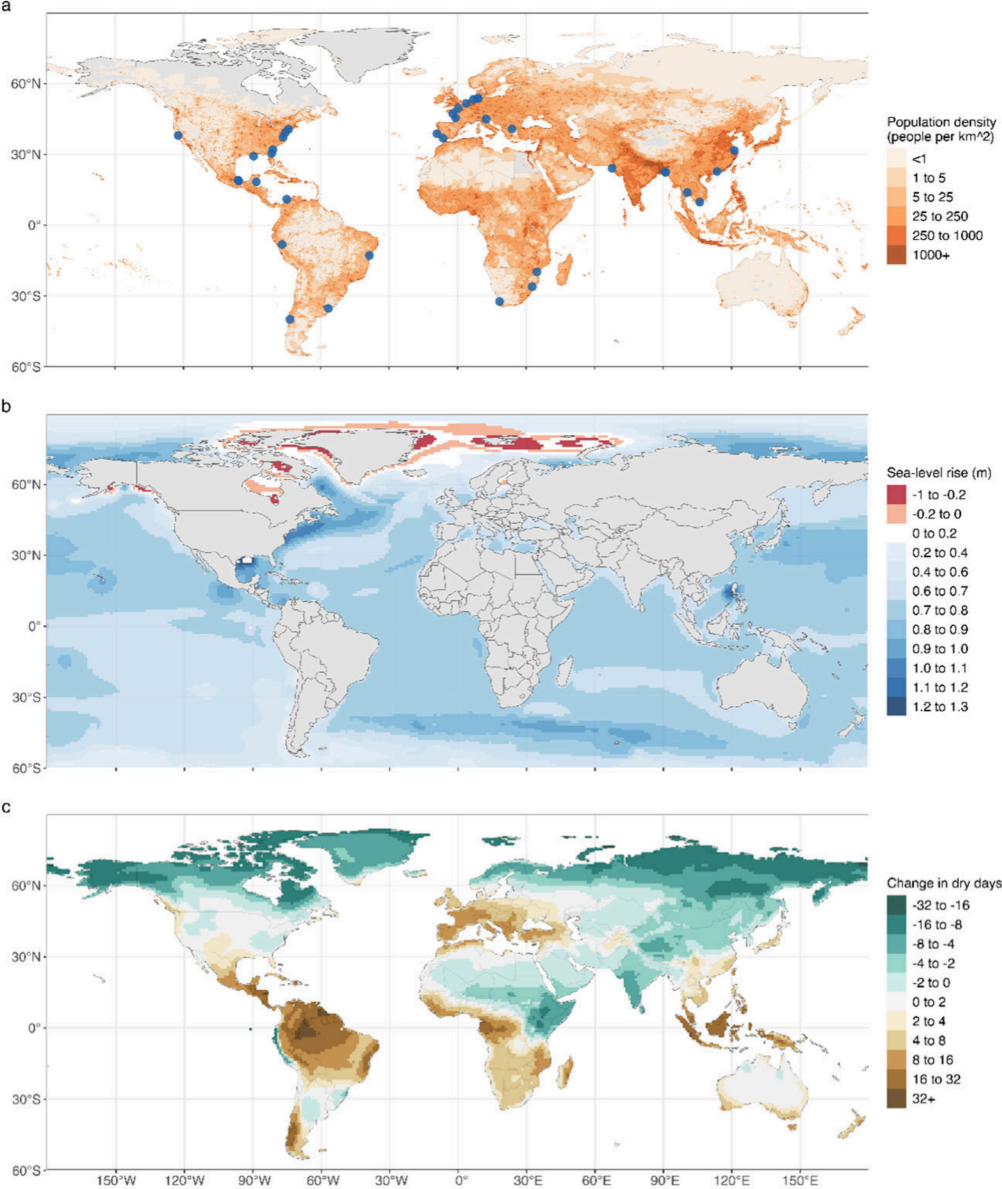
(a) Global map of population density (color) and locations of the tidal rivers with reported saltwater contamination issues (blue dots). Global maps of the projected (b) median regional relative sea-level change and (c) seasonal mean relative changes (%) in the number of dry days (i.e., days with less than 1 mm of rain) from 1995–2014 to 2100 averaged across available Coupled Model Intercomparison Project Phase 6 (CMIP6) models in the high emission SSP5-8.5 scenario. The sea-level projection is from ref 111 and the dry days projection is from ref 135.

The risk of salt contamination extends to uses other than drinking water, including thermoelectric power, agricultural irrigation, industrial production, mining, and aquaculture.^27^ Salt contamination of irrigation water damages conventional agricultural crops (e.g., corn and beans) and forces farmers to grow salt-tolerant crops (e.g., cotton and grain sorghum) that are less profitable.^28^ High salinity can be detrimental or even fatal to many freshwater finfish species while favoring salt-tolerant invasive species.^29^ High chloride concentration promotes galvanic corrosion of lead-bearing materials^30,31^ and pitting corrosion of copper.^32^ Along water distribution systems, elevated chloride concentration can increase mobilization of lead from pipes into drinking water.^33^ Critical transportation infrastructure, such as steel-reinforced concrete bridges, may also suffer from corrosion after an initiation period, in which the steel reinforcement becomes more vulnerable when the oxide layer is removed due to chloride exposure.^34^

## 2 A Multidimensional and Multidisciplinary Problem

A tidal river, located in the upper part of an estuary, is influenced by both river flows from land and tides from the ocean (Figure 2a). It is a vital but understudied nexus between hydrology and oceanography.^35,36^ Saltwater contamination of tidal rivers is a multidimensional and multidisciplinary problem involving physical and biogeochemical processes across the watershed–river–estuary–ocean continuum.

**Figure 2:**
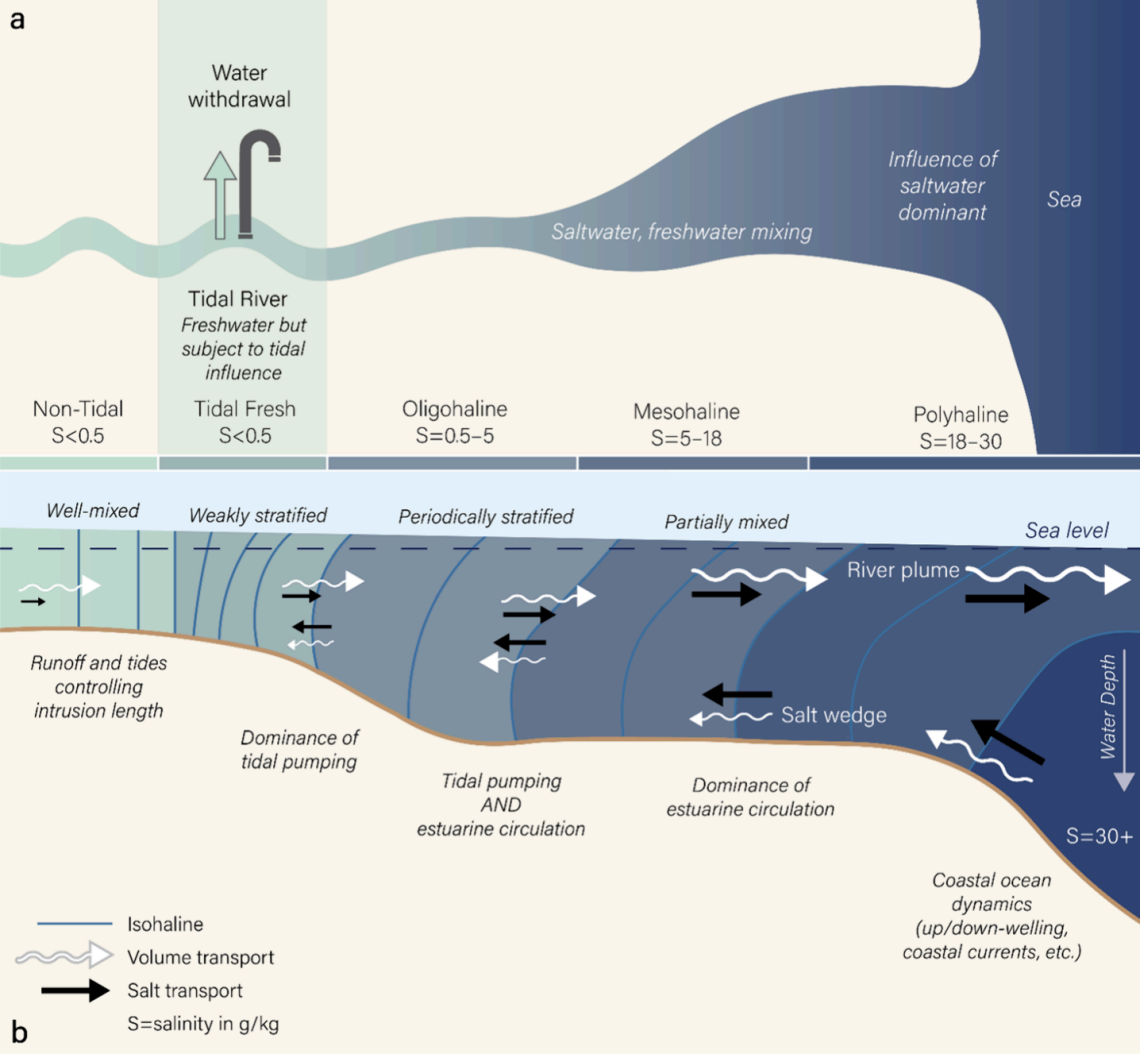
(a) A schematic plan view of an estuary showing different salinity (in units of g kg^–1^) subregions including the tidal river where many drinking water intakes are located. (b) A schematic along-channel section view of the typical volume and salt transport regimes in an estuary. Blue lines show isohalines, and colors show salinity. The white arrows indicate volume transports, while the black arrows indicate salt transports. The dotted line shows the position of the mean sea level. Note that for corresponding pairs of arrows, incoming and outgoing salt transports are almost identical, while outgoing volume transports are substantially larger than the incoming volume transports due to the river runoff.

### 2.1 Oceanic Saltwater Intrusion into Estuaries

Although the river flow transports water including salt seaward, other processes transport salt in the up-estuary direction. The two most important of those processes are estuarine circulation and tidal pumping (Figure 2b). Estuarine circulation is a bidirectional residual circulation with a near-bottom, up-estuary-directed current of increased salinity and a near-surface, down-estuary-directed current of fresher water, resulting in a vertically integrated up-estuary salt transport.^37^ Estuarine circulation^38,39^ is primarily due to the density gradient between salt and fresh water,^40^ but it may also be influenced by tidal straining,^41,42^ lateral circulation,^43^ and estuarine convergence.^44^ Tidal pumping is the vertically integrated temporal covariance of vertically averaged horizontal velocity and salinity, meaning that higher salinity during flood tide and lower salinity during ebb tide result in up-estuary salt transport.^45^ Generally, in relatively deep estuaries with weaker tides, estuarine circulation dominates, and in shallow estuaries with strong tides, tidal pumping dominates. In partially mixed estuaries, both processes may be of comparable magnitude.^46^

Since the down-estuary transport scales with the river flow, saltwater intrusion extends farther landward during droughts and shifts seaward during high flows. Effects of variations in tidal amplitude with the spring–neap cycle depend on the dominant salt transport mechanism, with estuarine circulation generally decreasing during periods with stronger tidal mixing, while tidal pumping increases with tidal amplitude. Thus, water depth, tidal amplitude, and river flows are the major processes influencing saltwater intrusion.^47–49^ Additionally, differences in saltwater intrusion length between estuaries are influenced by differences in morphology such as channel area or depth, curvature, channel–shoal geometry, branching, sills, constrictions, convergence, and much more.^50,51^

### 2.2 Oceanic and Estuarine Processes Influencing Saltwater Intrusion

The influences of river flow, tidal amplitude, and estuarine bathymetry on saltwater intrusion can be estimated from scaling based on the salt transport equation.^40,52^ In some estuaries like the Hudson River, the saltwater intrusion length *L* has been observed to scale as *Q* ^–1/3^, where *Q* is the river flow,^53,54^ but *L* is much less sensitive to river flow in other estuaries, such as the Delaware Bay^55–57^ and San Francisco Bay.^52^ In the Delaware Bay, both channel bathymetry and spring–neap variations in mixing contribute to the weak dependence of *L* on *Q*,^57,58^ while in the San Francisco Bay it has been attributed to influences of stratification on mixing and the along-channel variation in bathymetry.^52,59,60^ In partially mixed estuaries, *L* scales inversely with the tidal velocity *U*_*t*_ as strong vertical mixing limits the landward saltwater intrusion. In relatively shallow, well-mixed estuaries where tidal pumping dominates, however, *L* ∼ *Q* ^–1^ *U*_*t*_.^60^ These differing sensitivities of *L* to *Q* and *U*_*t*_ highlight the challenge in predicting saltwater intrusion.

Saltwater intrusion is highly sensitive to water depth *H*, as suggested in the scaling *L* ∼ *H* ^2^ from theory.^40^ Both sea-level rise and channel dredging can increase saltwater intrusion and tidal range.^61–64^ In most industrialized estuaries, dredging dominates other processes that increase the water depth. Channel deepening has been shown to increase saltwater intrusion and modify tidal amplitude in a number of estuaries worldwide.^63,65–67^ Even in wide estuaries where deepening of a narrow channel will only modestly increase channel cross-sectional area, the impact on salt flux is still significant because landward salt flux is focused in the deep channel.^68,57^

Saltwater intrusion is also influenced by coastal sea-level oscillations and the direct forcing of the wind on the estuary.^69,70^ Both the local and remote wind forcing drive a barotropic adjustment that produces transient landward salt fluxes reversing the river flow.^53,57,71,72^ Increases in saltwater intrusion with the passage of storm events can temporarily threaten water supplies, as seen in the Changjiang River^73^ and Delaware River.^74^

Increased offshore ocean salinity enhances the density contrast between river and oceanic water and, therefore, intensifies the estuarine circulation and saltwater intrusion. For example, the bottom salinity in the Chesapeake Bay covaries with the salinity in the Mid-Atlantic Bight on decadal time scales.^75–77^ Also, episodic events, such as upwelling and downwelling, can change the salinity of the inflowing oceanic water. An extreme example is the Western Baltic Sea, where the salinity outside the estuary can increase from 10 to 20 g kg^–1^ within a few hours.^78,79^ River plumes could also interact and thus change the salinity of the inflow waters.^80^

### 2.3 Hydrological Processes Influencing Freshwater Availability and Delivery

Transport of water and solutes such as salts in the watershed occurs through multiple hydrological pathways: surface runoff, soil percolation, subsurface lateral flow, groundwater flow, and river flow.^81^ The connectivity among these pathways is critical for understanding water transport in the watershed. Groundwater sustains about half of the river flow on average and is dominant during low-flow periods.^82^ Drought conditions can propagate across the hydrological pathways. A decline of precipitation beyond normal conditions reduces watershed soil moisture, which may, in turn, lead to reduced river flow through lower groundwater levels and decreases in groundwater contribution to the river flow. This drought cascade has led to the identification of different drought types:^83,84^ meteorological drought measured using precipitation, agricultural drought based on soil moisture, and hydrological drought measured using river flow or low-flow indicators.

The hydrological cycle is highly sensitive to the temperature. Warming increases evaporative demand and vegetation water use and, in cold climates, can lead to shifts in the partitioning between rain and snow.^85^ Increasing the level of evapotranspiration (surface evaporation and plant transpiration) can lead to soil drying. Besides reductions in soil moisture, drying is associated with reduced groundwater levels and baseflow, and the proportion of groundwater contributing to river flow.^85^ Changes in the partitioning of rain and snow can increase the early spring river flow at the expense of summer river flow. Rain-on-snow flooding events can result in freshwater pulses.

### 2.4 Freshwater Salinization and Secondary Effects on Water Quality and Ecosystems

Besides oceanic saltwater intrusion, salinity in the “fresh” end member of tidal rivers has increased due to numerous anthropogenic activities within watersheds, such as human-accelerated weathering, road salts for deicing, irrigation, and fertilizers.^25,86–88^ Urbanization and agricultural land use led to human-accelerated weathering of concrete impervious surfaces,^89^ which increases pH and concentrations of base cations.^89,90^ Due to the increased use of weathering agents and easily weathered substrates, the concentrations and loads of chemical weathering products such as alkaline salts and carbonates are increasing in rivers.^89,91–94^ For example, there have been increasing long-term trends in alkalinity and calcium concentration in approximately 2/3 of the major rivers draining the US East Coast^92^ and the seasonal impacts of salinization extend to tidal waters.^95^ It is also recognized that multiple ions (Ca^2+^, HCO_3_^–^, Mg^2+^, and K^+^) contribute to freshwater salinization.^89,94,96–98^

Freshwater salinization can lead to secondary effects, exacerbating hypoxia, mobilizing contaminants and affecting the distribution and abundance of species.^90,99–101^ Salinization can mobilize a wide variety of contaminants, including nutrients, metals, radionuclides, and arsenic.^90,102^ Pulses in salinity may trigger the release of many heavy metals from sediments and soils to streams and rivers that can persist for days and weeks.^89,97^ In addition, saltwater intrusion has been shown to enhance mobilization of phosphorus, arsenic, and other contaminants in groundwater.^90^ Recent research showed that salinization effects on contaminant mobilization extend to tidal rivers.^103^

### 2.5 Geographic Distribution and Use Types of Water Intakes

To assess the societal impacts of salinization, we need to identify and characterize the water intakes along tidal rivers. In the US, water intakes have been characterized in terms of use (public supply, irrigation, aquaculture, mining, domestic, livestock, industrial, and thermoelectric power), source (surface water or groundwater), salinity, amount of water withdrawn, and fraction of the withdrawal that is consumptive.^27^ As a first approximation of the uses of tidal rivers, we considered all surface freshwater withdrawals in the US in 2015, 96% of which were in four use types: thermoelectric power (48%), irrigation (31%), public supply (12%), and industrial (6%). For the thermoelectric power and industrial use types, saline water is also withdrawn. Therefore, the biggest impacts on human water use of tidal rivers are expected to be on irrigation and public supply.

None of the above water intake characterizations determine equivocally the tidal character of the water. As part of an ongoing study to identify water intakes on the Chesapeake Bay, we contacted water agencies within the two states that cover most of the Bay shoreline: Maryland and Virginia. The Maryland database contained 895 intakes, 130 of which were identified as tidal. Of those intakes, the use types were mainly agricultural irrigation (53%). The Virginia data set did not distinguish between tidal and nontidal intakes. The length of the Maryland shoreline is 6% of that of the contiguous US,^104^ so if Maryland is typical, then there may be as many as 2000 tidal water intakes in the US.

Figure 1a shows where saltwater intrusion into tidal rivers has impacted or is expected to impact irrigation and public water supply around the world. Some notable examples of impacts on irrigation from saltwater intrusion are the Ganges–Brahmaputra delta in Bangladesh,^105^ the Changjiang River in China,^106^ the Shatt-Al Arab River in Iraq,^107^ the Bay of Fundy in Canada,^108^ and South Kalimantan in Indonesia.^109^

### 2.6 Critical Need for Interdisciplinary Research

Currently, three research communities are working on different aspects of salt contamination in tidal rivers. Estuarine oceanographers have focused on salt transport in the mesohaline region of an estuary, whereas saltwater intrusion into the tidal river region may be controlled by different physical processes (Figure 2). Hydrologists are mostly concerned with the occurrence of floods and droughts, and they have long ignored tides and their interactions with river networks. Biogeochemists studying freshwater salinization have mostly focused on nontidal rivers. To address the issue of salt contamination of water supplies in tidal rivers, convergent research that integrates these communities is needed.

## 3 Climate Change as a Major Driver of Saltwater Intrusion

The frequent reports of salt contamination of water supplies in recent years point to climate change as a major driver of saltwater intrusion into tidal rivers. Although the underlying mechanisms are not yet well understood, recent research has highlighted the role of several processes, including accelerated relative sea-level rise, changing drought and river flow regimes, and extreme weather events.

### 3.1 Impacts of Sea-Level Rise and Changing Ocean Circulation

Sea level rose ∼0.2 m during the 20th century^110^ and is projected to increase ∼1 m by the end of the 21st century,^111^ but there are large regional variations in the sea-level rise rate due to Earth’s uneven gravity field, glacial isostatic adjustment and ocean dynamics (Figure 1b).^112^ Sea-level rise increases saltwater intrusion into estuaries. Analysis of historical data in the Chesapeake and Delaware Bays showed a clear connection between sea-level rise and estuarine salinity increases.^76,113^ In the San Francisco Bay and the James River the effects of sea-level rise were found to be stronger during periods of low river flow.^114,115^ Climate change increases the risk of extreme saltwater intrusion across European estuaries, including the Loire, Scheldt, Rhine–Meuse, Elbe, and Humber estuaries.^116^ In Asia sea-level rise is a major factor enhancing saltwater intrusion into the Changjiang, Pearl, Mekong, Gorai, and Ganges Rivers.^117,118^ A recent study of 18 estuaries worldwide suggests that future climate change would increase estuarine salt intrusion mainly through sea-level rise rather than through reduced river flow.^119^ The effects of sea-level rise may be cast as an increase in the mean water depth of the estuary. Both salt flux and saltwater intrusion length increase with the depth to the second or third power, depending on the details of how mixing is modified by the increased water depth.^120,121^

It is important to note that many estuaries are capable of rapid morphological change such that the mean depth of an estuary may increase more slowly, or not at all, with sea-level rise due to sediment accumulation. The estuarine circulation that drives landward salt flux also promotes trapping of fine sediment from both riverine and marine sources.^122^ Near-bottom residual currents transport sediment landward into the estuary, and the strong feedback among channel cross-sectional area, tidal currents, bed shear stress, and sediment erosion and deposition results in estuaries maintaining morphological equilibrium depths.^123^ Given sufficient sediment supply, estuaries tend to accrete vertically at rates similar to the relative sea-level rise.^124,125^ Consequently, the response of saltwater intrusion may be muted relative to the nonlinear scaling *L* ∼ *H* ^2^.

Saltwater intrusion could also be driven by rising coastal sea levels due to changing ocean circulation or warming. The accelerated sea-level rise along the US east coast north of Cape Hatteras during 1950–2009 was attributed to the weakening of Atlantic Meridional Overturning Circulation and the Gulf Stream,^126,127^ whereas the rapid sea-level rise in the US southeast and Gulf coast in recent years was thought to be either associated with stereodynamic effects due to warming of coastal currents^128^ or amplified by internal climate variability in the tropical North Atlantic.^129^ Significant correlation has been found between El Niño–Southern Oscillation and extreme sea levels across the Pacific,^130^ including the west coast of South and North America^131,132^ and the South China Sea.^133^

### 3.2 Impacts of Changing Hydrological Cycle and Competing Water Uses

Hydroclimatic shifts, such as increased drought severity, affect all of the continents (Figure 1c). The Mediterranean Sea region, southeastern Africa, parts of Central and South America, and Indonesia could experience significant increases in the number of dry days per year by the end of this century.^134,135^ Climate model projections indicate that drought risk will increase, with changes varying across regions, seasons, and drought characteristics (e.g., drought onset, severity, and duration).^136,137^ In high northern latitudes and high-elevation areas of the midlatitudes, climate projections show a consistent decline in river flow, an indicator of hydrological drought, in the summer months due to warming impacts on precipitation and changes in snow dynamics (snowpack melts earlier in the season).^138^ In other regions, river flow declines are closely associated with decreased precipitation patterns, such as those in regions with Mediterranean climates.

Coastal water supplies are threatened by compounding stressors, including the challenge of balancing competing needs for freshwater resources. Coastal population growth increases needs not only for water supplies but also for energy, infrastructure, and urban space. For example, maintaining supplies for increasing water needs (municipal, agricultural, etc.) might require shifts to groundwater aquifers or to desalination, both of which have a higher energy burden than surface water supplies.

### 3.3 Impacts of Increasing Climate Extremes

Although saltwater intrusion is affected by long-term trends in river flow and water depth, salinity spikes at water intakes typically occur over a short period and may be affected by a flash drought or short-term sea-level variability, such as from storm surge.^139^ Extreme sea levels may occur more frequently due to secular sea-level rise and an increase in intensity or frequency of storms.^111,140^ Despite an overall decline in the number of tropical cyclones,^141^ several findings suggest conditions that would increase the variability of coastal sea level^142^ (and, by inference, salinity), including increases in major hurricanes^143–145^ and the number of landfalling tropical cyclones.^146^

Variability in river flow is also likely to increase from daily to interannual time scales due to increases in heavy precipitation^135^ and extreme drought. At temperate latitudes, river flow is highest during the winter and spring and lowest during the summer and fall, but climate change is expected to increase winter and spring precipitation, with an increasing fraction of that precipitation as liquid.^135^ While summer and fall precipitation projections are more variable, warming will increase evapotranspiration, which will reduce river flow and enhance saltwater intrusion. Hence, we can expect the amplitude of the annual cycle in river flow to increase in the future.

## 4 Recommendations for Future Research and Development

The above synthesis reveals a critical need for convergent interdisciplinary research that must be integrated across oceanography, hydrology, and water resource management. We identify several key topics requiring immediate attention and propose a research agenda for developing a decision support tool to manage salt contamination of water supplies in tidal rivers, as outlined below.

### 4.1 Ion-Specific Measurements

The relative proportions of dissolved salts differ between seawater and nontidal riverine water.^147^ Consequently, specific conductivity meters cannot be used to infer the salinity of tidal rivers.^148^ Major ions, such as sodium and calcium, can vary by an order of magnitude among rivers.^149,25^ To characterize salt contamination in tidal rivers, we need to measure concentrations of major salt ions and enhance monitoring. These measurements will expand our limited understanding of the sources, transport, and fate of major salt ions over watersheds and in tidal rivers. Some salt ions, such as Na^+^ and Cl^–^, behave conservatively, whereas other salt ions, such as Ca^2+^ and Mg^2+^, may experience changes in solubility. Other ions, such as carbonates, have been increasing in rivers^93,149^ but may be influenced by biological generation and biological uptake.^150^ In addition, the combined use of conductivity and pH measurements may be useful as proxies in predicting the behavior of nonconservative ions or shifts in ion sources with changing hydrology.^97^

### 4.2 Development of Ion-Specific Hydrological–Hydrodynamic Models

Coupled hydrological and hydrodynamic models are used to predict compound flooding^151,152^ and can be extended to predict salt transport. Given the salt composition difference between riverine water and seawater, we need hydrodynamic models that track not only the salinity but also the concentrations of individual salt ions. The salinity module recently incorporated into the Soil and Water Assessment Tool (SWAT) has demonstrated the capability to simulate salt transport in all major hydrologic pathways at the watershed scale and capture important solution reaction chemistry.^81^ The SWAT+ salt module simulates eight major salt ions (Na^+^, Cl^–^, Mg^2+^, K^+^, Ca^2+^, CO_3_^2–^, HCO_3_^–^, and SO_4_^2–^), which fortunately includes the top seven (all but CO_3_^2–^) ions in seawater by weight. Some of these ions (e.g., Na^+^) are conservative and can be modeled as passive tracers. Other ions (e.g., Ca^2+^) are nonconservative, but recent progress in carbonate chemistry modeling could help predict these ions.^153–157^ The standard seawater equation of state also needs to be modified for calculating water density in tidal rivers.^158^

### 4.3 Salinity Management Strategies Informed by Mechanistic Models and AI Algorithms

Climate adaptation and water plans reveal many different implementations of salinity management strategies, ranging in expense and complexity.^159^ For drinking water systems, desalination may seem like an obvious strategy, but it requires large up front capital expenditures^160^ and is expensive to operate and maintain.^161^ Managing flow releases from reservoirs may protect coastal water users from increasing salinity at a relatively lower cost. This method has been in use in the Delaware River and Hudson River basins and elsewhere.^19,162^

Climate adaptations, such as reservoir releases, are often supported by optimization methods and used to tailor releases to short- and long-term projections of regional hydroclimatic conditions.^163^ These operations are affected by a “cascade of uncertainties”^164^ that significantly affect our ability to quantify the expected effectiveness of adaptive responses.^165^ Several methods have been advanced to support dynamically adaptive planning and operations.^166^ State-of-the-art reservoir operation methods utilize tools from closed-loop control and multiobjective optimization to design operational policies that meet multiple goals by responding to dynamic conditions.^167,163^ These approaches have recently evolved to the use of multiobjective reinforcement learning, a type of machine learning where Artificial Intelligence (AI) agents learn to make decisions by receiving rewards or penalties for their actions. The goal is to train adaptive policies that can meet diverse and conflicting operational goals, by exposing them to a wide range of dynamic conditions.^165,168,169^ As such, these policies can be trained to also consider salinity mitigation goals in tidal rivers with inland reservoirs,^170^ taking into account seasonal variability and long-term changes in hydroclimatic conditions so that dynamic salinity dilution needs can be met.
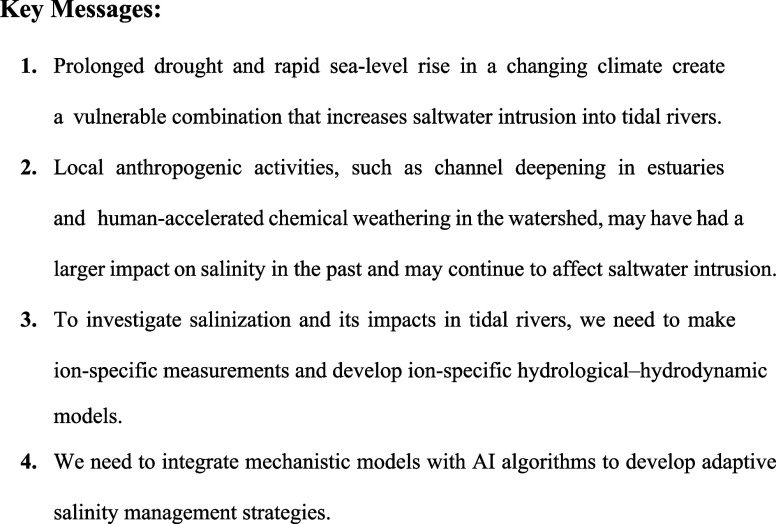


### 4.4 Developing a Decision Support Tool Using a Human-Centered Design

By integrating ion-specific hydrological–oceanographic models with AI-based optimization algorithms, we recommend the development of a decision support tool for predicting and managing salt contamination of water supplies in tidal rivers, as illustrated in Figure S3. The model predictions must be evaluated against enhanced real-time monitoring of conditions in tidal rivers, including ion-specific measurements. There is a wide range of stakeholders and potential users, ranging from regulators and water resource managers at local, state, federal, and intergovernmental agencies to stakeholders from the public water supply, agricultural, industrial, power generation, and environmental sectors. They may have different goals, such as short-term management strategies (e.g., reservoir releases) and long-term planning decisions (adaptative policy pathways). To develop a decision support system that can meet user needs, it is important to apply human-centered design and engage with stakeholders during all phases of software development.

## Supplementary Material



## Notes

Sample time series of conductivity at tidal rivers and a schematic diagram for a salinity management decision support system. (PDF)

## References

[1] Hallenbeck W. H., Brenniman G. R., Anderson R. J. (1981). High sodium in drinking water and
its effect on blood pressure. Ameri. J. Epidem..

[2] Calabrese E. J., Tuthill R. W. (1981). The influence of elevated levels of sodium in drinking
water on elementary and high school students in Massachusetts. Sci. Total Environ..

[3] Vineis P., Chan Q., Khan A. (2011). Climate change impacts
on water salinity
and health. J. Epide. Glo. Hea..

[4] Khan A. E., Ireson A., Kovats S., Mojumder S. K., Khusru A., Rahman A., Vineis P. (2011). Drinking water
salinity and maternal
health in coastal Banglaldesh: implications of climate change. Environ. Health Perspect.

[5] US Army Corps of Engineers . https://www.mvn.usace.army.mil/Media/News-Releases/Article/3544528/usace-begins-barging-fresh-river-water-to-plaquemines-parish-water-treatment-fa/. (accessed 2024-11-24).

[6] California DWR (Department of Water Resources) . Construction Begins on Emergency Drought Barrier in Sacramento-San Joaquin Delta. https://water.ca.gov/News/News-Releases/2021/June-21/Emergency-Drought-Barrier-Construction-Delta. (accessed 2024-11-24).

[7] Reuters . 2020. Salty water in Bangkok is new ’reality’ as sea pushes farther inland. https://www.reuters.com/article/world/salty-water-in-bangkok-is-new-reality-as-sea-pushes-farther-inland-idUSKBN1Z90V2/#:~:text=Bangkok’s%20water%20authority%20said%20the,water%20by%20taking%20shorter%20showershttps://www.reuters.com/article/world/salty-water-in-bangkok-is-new-reality-as-sea-pushes-farther-inland-idUSKBN1Z90V2/#:~:text=Bangkok’s%20water%20authority%20said%20the,water%20by%20taking%20shorter%20showers. (accessed 2024-11-24).

[8] New York Times . 2022. https://www.nytimes.com/2022/10/10/climate/netherlands-drought-climate-change.html (accessed 2024-11-24).

[9] Lassiter A. (2021). Rising seas,
changing salt lines, and drinking water salinization. Curr. Opin. Envi. Sust..

[10] Hoguane A. M., Gammelsrød T., Mazzilli S., Antonio M. H., da Silva N. B. F. (2020). The
hydrodynamics of the Bons Sinais Estuary: The value of simple hydrodynamic
tidal models in understanding circulation in estuaries of central
Mozambique. Reg. Stud. Mar. Sci..

[11] Bellafiore D., Ferrarin C., Maicu F., Manfe G., Lorenzetti G., Umgiesser G., Zaggia L., Valle Levinson A. (2021). Saltwater
intrusion in a Mediterranean delta under a changing climate. J. Geophys. Res. Oceans.

[12] Schmidt, S. A 14-year multi-sites and high-frequency monitoring of salinity in the tidal Garonne River (S-W France) reveals marked interannual variability in marine intrusion. In Nguyen, K. , Guillou, S. , Gourbesville, P. , Thiébot, J. , Eds.; Estuaries and coastal zones in times of global change; Springer Water. Springer. 2020, 10.1007/978-981-15-2081-5_1.

[13] Kolb P., Zorndt A., Burchard H., Gräwe U., Kösters F. (2022). Modelling the impact of anthropogenic measures on saltwater
intrusion in the Weser estuary. Ocean Sci..

[14] Zhu J., Cheng X., Li L., Wu H., Gu J., Lyu H. (2020). Dynamic mechanisms of an extremely
severe saltwater intrusion in
the Changjiang Estuary in February 2014. HESS.

[15] Payo-Payo M., Bricheno L. M., Dijkstra Y. M., Cheng W., Gong W., Amoudry L. O. (2022). Multiscale temporal response of salt intrusion to transient
river and ocean forcing. J. Geophy. Res.: Oceans.

[16] Bricheno L. M., Wolf J., Sun Y. (2021). Saline intrusion
in the Ganges-Brahmaputra-Meghna
megadelta. Estuar. Coast. Shelf Sci..

[17] Garcés-Vargas J., Schneider W., Pinochet A., Piñones A., Olguin F., Brieva D., Wan Y. (2020). Tidally forced saltwater
intrusions might impact the quality of drinking water, the Valdivia
River (40° S), Chile estuary case. Water.

[18] Ospino S., Restrepo J. C., Otero L., Pierini J., Alvarez-Silva O. (2018). Saltwater
intrusion into a river with high fluvial discharge: a microtidal estuary
of the Magdalena River, Colombia. J. Coast.
Res..

[19] Hoagland P., Beet A., Ralston D., Parsons G., Shirazi Y., Carr E. (2020). Salinity intrusion
in a modified river-estuary system: an integrated
modeling framework for source-to-sea management. Front. Mar. Sci..

[20] Diffenbaugh N. S., Swain D. L., Touma D. (2015). Anthropogenic
warming has increased
drought risk in California. Proc. Natl. Acad.
Sci. U.S.A..

[21] Mukherjee S., Mishra A., Trenberth K. E. (2018). Climate change and drought: a perspective
on drought indices. Curr. Clim. Chan. Rep..

[22] Ford T. W., Otkin J. A., Quiring S. M., Lisonbee J., Woloszyn M., Wang J., Zhong Y. (2023). Flash drought
indicator intercomparison
in the United States. J. Appl., Meteo. Clim..

[23] Lesinger K., Tian D. (2022). Trends, variability, and drivers of flash droughts in the contiguous
United States. Water Resour. Res..

[24] Walker D. W., Vergopolan N., Cavalcanter L., Smith K. H., Agoungbome S. M. D., Almagro A., Apurv T., Dahal N. M., Hoffman D., Singh V., Xiang Z. (2024). Flash drought typologies and societal
impacts: a worldwide review of occurrence, nomenclature, and experience
of local populations. Wea., Clim., Soc..

[25] Kaushal S. S., Groffman P. M., Likens G. E., Belt K. T., Stack W. P., Kelly V. R., Band L. E., Fisher G. T. (2018). Increased salinization
of fresh water in the northeastern United States. Proc. Natl. Acad. Sci. U.S.A..

[26] Beibei E., Zhang S., Driscoll C. T., Wen T. (2023). Human and natural impacts
on the US freshwater salinization and alkalinization: A machine learning
approach. Sci. Total Environ..

[27] Dieter C. A., Maupin M. A., Caldwell R. R., Harris M. A., Ivahnenko T. I., Lovelace J. K., Barber N. L., Linsey K. S. (2018). Estimated use of
water in the United States in 2015. U.S. Geological
Survey Circular.

[28] Yamaguchi T., Blumwald E. (2005). Developing salt-tolerant
crop plants: challenges and
opportunities. Trends Plant Sci..

[29] Kujawa R., Piech P. (2022). Influence of water salinity on the growth and survivability of Asp
Larvae *Leuciscus aspius* (Linnaeus, 1758) under controlled
conditions. Animals.

[30] Ng D.-Q., Lin Y.-P. (2016). Evaluation of lead release in a simulated lead-free
premise plumbing system using a sequential sampling approach. Int. J. Environ. Res. Public Health..

[31] Willison H., Boyer T. H. (2012). Secondary effects
of anion exchange on chloride, sulfate,and
lead release: systems approach to corrosion control. Water Res..

[32] Schock, M. R. , Lytle, D. A. Internal corrosion and deposition control. In Edzwald, J. K. , Ed.; Water Quality and Treatment: A Handbook on Drinking Water.; McGraw-Hill, New York, NY, 2011; pp 20.21–20.103.

[33] Stets E. G., Lee C. J., Lytle D. A., Schock M. R. (2018). Increasing chloride
in rivers of the conterminous U.S. and linkages to potential corrosivity
and lead action level exceedances in drinking water. Sci. Total Environ..

[34] Enright M. P., Frangopol D. M. (1998). Probabilistic
analysis of resistance degradation of
reinforced concrete bridge beams under corrosion. Eng. Struct..

[35] Moldwin, M. Tidal river dynamics, Eos 2016, 97, 10.1029/2018EO049541.

[36] Hoitink A. J. F., Jay D. A. (2016). Tidal river dynamics:
implications for deltas. Rev. Geophys..

[37] Geyer W. R., MacCready P. (2014). The estuarine
circulation. Annu.
Rev. Fluid Mech..

[38] Burchard H., Hetland R. D., Schulz E., Schuttelaars H. M. (2011). Drivers
of residual estuarine circulation in tidally energetic estuaries:
Straight and irrotational channels with parabolic cross section. J. Phys. Oceanogr..

[39] Burchard H., Schulz E., Schuttelaars H. M. (2014). Impact
of estuarine convergence on
residual circulation in tidally energetic estuaries and inlets. Geophys. Res. Lett..

[40] Hansen D. V., Rattray M. (1965). Gravitational circulation in straits
and estuaries. J. Mar. Res..

[41] Simpson J. H., Brown J., Matthews J., Allen G. (1990). Tidal straining,
density
currents, and stirring in the control of estuarine stratification. Estuaries.

[42] Jay D. A., Musiak J. D. (1994). Particle trapping in estuarine tidal flows. J. Geophys. Res. Oceans.

[43] Lerczak J. A., Geyer R. W. (2004). Modeling the lateral
circulation in straight, stratified
estuaries. J. Phys. Oceanogr..

[44] Ianniello J. P. (1979). Tidally
induced residual currents in estuaries of variable breadth and depth. J. Phys. Oceanogr..

[45] Geyer, W. R. , Nepf, H. Tidal pumping of salt in a moderately stratified estuary. In Buoyancy Effects on Coastal and Estuarine Dynamics. Coastal and Estuarine Studies; Aubrey, D. G. , Friedrichs, C. T. , Ed.; 1996; Vol. 53, pp 213–226.

[46] Hendrickx G. G., Antolínez J. A., Herman P. M. (2023). Predicting the response of complex
systems for coastal management. Coast. Eng..

[47] Díez-Minguito M., Contreras E., Polo M. J., Losada M. A. (2013). Spatio-temporal
distribution, along-channel transport, and post-river flood recovery
of salinity in the Guadalquivir estuary (SW Spain). J. Geophys. Res. Oceans..

[48] Becherer J., Flöser G., Umlauf L., Burchard H. (2016). Estuarine circulation
versus tidal pumping: Sediment transport in a well-mixed tidal inlet. J. Geophys. Res. Oceans..

[49] Garcia A. M. P., Geyer W. R., Randall N. (2022). Exchange flows
in tributary creeks
enhance dispersion by tidal trapping. ESCO.

[50] Garcia A. M. P., Geyer W. R. (2023). Tidal dispersion in short estuaries. J. Geophys. Res. Oceans..

[51] Hendrickx G. G., Manuel L. A., Pearson S. G., Aarninkhof S. G., Meselhe E. A. (2024). An earthen sill as a measure to mitigate
salt intrusion
in estuaries. ESCO.

[52] Monismith S. G., Kimmerer W., Burau J. R., Stacey M. T. (2002). Structure and flow-induced
variability of the subtidal salinity field in northern San Francisco
Bay. J. Phys. Oceanogr..

[53] Lerczak J. A., Geyer W. R., Chant R. J. (2006). Mechanisms
driving the time-dependent
salt flux in a partially stratified estuary. J. Phys. Oceanogr..

[54] Lerczak J. A., Geyer W. R., Ralston D. K. (2009). The temporal
response of the length
of a partially stratified estuary to changes in river flow and tidal
amplitude. J. Phys. Oceanogr..

[55] Garvine R. W., Wong K.-C. (1992). The axial salinity
distribution in the Delaware estuary
and its weak response to river discharge. Estuar.
Coast. Shelf Sci..

[56] MacCready P. (2007). Estuarine
adjustment. J. Phys. Oceanogr..

[57] Aristizabal M., Chant R. (2013). A numerical study of
salt fluxes in Delaware Bay estuary. J. Phys.
Oceanogr..

[58] Aristizábal M. F., Chant R. J. (2015). An observational study of salt fluxes in Delaware Bay. J. Geophys. Res.-Oceans..

[59] Ralston D. K., Geyer W. R., Lerczak J. A. (2008). Subtidal
salinity and velocity in
the Hudson River estuary: observations and modeling. J. Phys. Oceanogr..

[60] MacCready P. (2004). Toward a unified
theory of tidally-averaged estuarine salinity structure. Estuaries.

[61] Holleman R. C., Stacey M. T. (2014). Coupling of sea level rise, tidal amplification, and
inundation. J. Phys. Oceanogr..

[62] Lee, S. N. , Li, M. , Zhang, F. Impact of sea-level rise on tidal ranges in Chesapeake and Delaware Bays. J. Geophys. Res. Oceans 2017, 122, 3917 10.1002/2016JC012597.

[63] Ralston D. K., Geyer W. R. (2019). Response to channel deepening of the salinity intrusion,
estuarine circulation, and stratification in an urbanized estuary. J. Geophys. Res. Oceans.

[64] Talke S. A., Familkhalili R., Jay D. A. (2021). The influence of channel deepening
on tides, river discharge effects, and storm surge. J. Geophys. Res.-Oceans.

[65] Pareja-Roman L. F., Chant R. J., Sommerfield C. K. (2020). Impact
of historical channel deepening
on tidal hydraulics in the Delaware Estuary. J. Geophys. Res. Oceans.

[66] de
Jonge V. N., Schuttelaars H. M., van Beusekom J. E., Talke S. A., de Swart H. E. (2014). The influence of channel deepening
on estuarine turbidity levels and dynamics, as exemplified by the
Ems estuary. Estuar. Coast. Shelf Sci..

[67] Talke S. A., Jay D. A. (2020). Changing tides:
The role of natural and anthropogenic
factors. Annu. Rev. Mar Sci..

[68] Geyer W. R., Ralston D. K., Chen J. L. (2020). Mechanisms
of exchange flow in an
estuary with a narrow, deep channel and wide, shallow shoals. J. Geophys. Res. Oceans.

[69] Wang D.-P. (1979). Subtidal
sea level variations in the Chesapeake Bay and relations to atmospheric
forcing. J. Phys. Oceanogr..

[70] Wong K.-C., Wilson R. E. (1984). Observations of
low-frequency variability in Great
South Bay and relations to atmospheric forcing. J. Phys. Oceanogr..

[71] Chen S., Sanford L. P. (2009). Axial wind effects
on stratification and longitudinal
salt transport in an idealized, partially mixed estuary. J. Phys. Oceanogr..

[72] Li Y., Li M. (2011). Effects of winds on stratification and circulation in a partially
mixed estuary. J. Geophys. Res.Oceans.

[73] Li L., Wang C., Pareja-Roman L. F., Zhu J., Chant R. J., Wang G. (2022). Effects of typhoon on saltwater intrusion
in a high discharge estuary. J. Geophys. Res.
Oceans.

[74] Cook S. E., Warner J. C., Russell K. L. (2023). A numerical investigation of the
mechanisms controlling salt intrusion in the Delaware Bay estuary. Estua., Coast. Shelf Sci..

[75] Mountain D. G. (2003). Variability
in the properties of shelf water in the Middle Atlantic Bight, 1977–1999. J. Geophys. Res. Oceans.

[76] Hilton, T. W. , Najjar, R. G. , Zhong, L. , Li, M. Is there a signal of sea-level rise in Chesapeake Bay salinity? J. Geophys. Res. 2008, 113, 10.1029/2007JC004247.

[77] Lee Y., Lwiza K. M. M. (2008). Factors driving bottom salinity variability in the
Chesapeake Bay. Cont. Shelf Res..

[78] Lange X., Klingbeil K., Burchard H. (2020). Inversions of estuarine circulation
are frequent in a weakly tidal estuary with variable wind forcing
and seaward salinity fluctuations. J. Geophys.
Res. Oceans.

[79] Burchard H., Klingbeil K., Lange X., Li X., Lorenz M., MacCready P., Reese L. (2025). The relation between exchange flow
and diahaline mixing in estuaries. J. Phys.
Oceanogr..

[80] Flöser G., Burchard H., Riethmüller R. (2011). Observational evidence for estuarine
circulation in the German Wadden Sea. Cont.
Shelf Res..

[81] Bailey R. T., Tavakoli-Kivi S., Wei X. (2019). A salinity module for SWAT to simulate
salt ion fate and transport at the watershed scale. Hydrol. Earth Syst. Sci..

[82] Xie J., Liu X., Jasechko S., Berghuijs W. R., Wang K., Liu C., Reichstein M., Jung M., Koirala S. (2024). Majority of global
river flow sustained by groundwater. Nat. Geosci..

[83] Mishra A. K., Singh V. P. (2010). A review of drought
concepts. J. Hydrol..

[84] Van
Loon A. F., Van Lanen H. A. J. (2012). A process-based typology of hydrological
drought. Hydrol. Earth Syst. Sci..

[85] Cook B. I., Mankin J. S., Marvel K., Williams A. P., Smerdon J. E., Anchukaitis K. J. (2020). Twenty-first
century drought projections in the CMIP6
forcing scenarios. Earth’s Futur..

[86] Cañedo-Argüelles M., Kefford B. J., Piscart C., Prat N., Schäfer R. B., Schulz C. J. (2013). Salinisation of rivers: an urgent ecological issue. Environ. Pollut..

[87] Thorslund J., Bierkens M. F., Oude Essink G. H., Sutanudjaja E. H., van Vliet M. T. (2021). Common irrigation drivers of freshwater
salinisation
in river basins worldwide. Nature Commun..

[88] Kaushal S. S., Mayer P. M., Likens G. E., Reimer J. E., Maas C. M., Rippy M. A., Grant S. B., Hart I., Utz R. M., Shatkay R. R., Wessel B. M. (2023). Five state
factors control progressive
stages of freshwater salinization syndrome. Limnol. Oceanogr. Lett..

[89] Kaushal S. S., Duan S., Doody T. R., Haq S., Smith R. M., Johnson T. A. N., Newcomb K. D., Gorman J., Bowman N., Mayer P. M., Wood K. L. (2017). Human-accelerated
weathering increases
salinization, major ions, and alkalinization in fresh water across
land use. Appl. Geochem..

[90] Kaushal, S. S. , Mayer, P. M. , Shatkey, R. R. , Maas, C. M. , Cañedo-Argüelles, M. , Hintz, W. D. , Wessel, B. M. , Tully, K. G. , Rippy, M. A. , Grant, S. B. Salinization of inland waters. 3rd ed. of Treatise on Geochemistry 2024. Elsevier.

[91] Barnes R. T., Raymond P. A. (2009). The contribution of agricultural
and urban activities
to inorganic carbon fluxes within temperate watersheds. Chem. Geol..

[92] Kaushal S. S., Likens G. E., Utz R. M., Pace M. L., Grese M., Yepsen M. (2013). Increased river alkalinization
in the Eastern US. Environ. Sci. Technol..

[93] Stets E. G., Kelly V. J., Crawford C. G. (2014). Long-term
trends in alkalinity in
large rivers of the conterminous US in relation to acidification,
agriculture, and hydrologic modification. Sci.
Total Environ..

[94] Bird D. L., Groffman P. M., Salice C. J., Moore J. (2018). Steady-state land cover
but non-steady-state major ion chemistry in urban streams. Environ. Sci. Technol..

[95] Kaushal S. S., Shelton S. A., Mayer P. R., Kellmayer B., Utz R. M., Relmer J. E., Baljunas J. B., Bhide S. V., Mon A., Rodriguez-Cardona B. M., Grant S. H., Newcomer-Johnson T. A., Malin J. T., Shatkay R. R., Collison D. C., Papageorgiou K., Escobar J., Rippy M., Likens G. R., Najjar R. G., Mejia A. I., Lassiter A., Li M., Chant R. J. (2025). Freshwater
faces a warmer, saltier, and alkaline future: 10 risks from climate
change, saltwater intrusion, and chain reactions. Biogeochemistry.

[96] Kaushal S. S., Likens G. E., Mayer P. M., Shatkay R. R., Shelton S. A., Grant S. B., Utz R. M., Yaculak A. M., Maas C. M., Reimer J. E., Bhide S. V. (2023). The anthropogenic
salt cycle. Nat. Rev. Earth Environ..

[97] Kaushal S. S., Likens G. E., Pace M. L., Haq S., Wood K. L., Galella J. G., Morel C., Doody T. R., Wessel B., Kortelainen P., Räike A. (2019). Novel ‘chemical cocktails’
in inland waters are a consequence of the freshwater salinization
syndrome. Philos. Trans. R. Soc. B.

[98] Rossi M. L., Kremer P., Cravotta C. A., III, Seng K. E., Goldsmith S. T. (2023). Land development
and road salt usage drive long-term changes in major-ion chemistry
of streamwater in six exurban and suburban watersheds, southeastern
Pennsylvania, 1999–2019. Front. Environ.
Sci..

[99] Kefford B. J., Buchwalter D., Cañedo-Argüelles M., Davis J., Duncan R. P., Hoffmann A., Thompson R. (2016). Salinized
rivers: degraded systems or new habitats for salt-tolerant faunas?. Biol. Lett..

[100] Velasco J., Gutiérrez-Cánovas C., Botella-Cruz M., Sánchez-Fernández D., Arribas P., Carbonell J. A., Millán A., Pallarés S. (2019). Effects of salinity changes on aquatic organisms in
a multiple stressor context. Philos. Trans.
R. Soc. B.

[101] Berger E., Frör O., Schäfer R. B. (2019). Salinity
impacts on river ecosystem processes: a critical mini-review. Philos. Trans. R. Soc. B.

[102] Lazur A., VanDerwerker T., Koepenick K. (2020). Review of
implications of road salt use on groundwater quality—corrosivity
and mobilization of heavy metals and radionuclides. WAPLAC.

[103] Teuchies J., De Deckere E., Bervoets L., Meynendonckx J., Van Regenmortel S., Blust R., Meire P. (2008). Influence of tidal
regime on the distribution of trace metals in a contaminated tidal
freshwater marsh soil colonized with common reed (Phragmites australis). Environ. Pollut..

[104] NOAA Shoreline . https://shoreline.noaa.gov/ (accessed on 2024-11-24).

[105] AI Masud M. M., Gain A. K., Azad A. K. (2020). Tidal river management
for sustainable agriculture in the Ganges-Brahmaputra delta: Implication
for land use policy. Land Use Policy.

[106] Chen X., Zong Y. (1999). Major impacts of sea-level
rise on
agriculture in the Yangtze delta area around Shanghai. Appl. Geogr..

[107] Abdullah A. D., Karim U. F. A., Masih I., Popescu I., Van der Zaag P. (2016). Anthropogenic and tidal influences on salinity levels
of the Shatt al-Arab River, Basra, Iraq. JRBM.

[108] Zhao Q., Chen Y., Gone K. P., Wells E., Margeson K., Sherren K. (2023). Modelling cultural ecosystem services
in agricultural dykelands and tidal wetlands to inform coastal infrastructure
decisions: A social media data approach. Mar.
Policy.

[109] Wasita W., Mansyur S., Hindarto I., Sunarningsih S., Susilawati S., Saptono N., Sujarwo W. (2024). Tidal rice farming
in South Kalimantan: tradition, advantages, and challenges. IJTK.

[110] Church J. A., White N. J., Aarup T., Wilson W. S., Woodworth P. L., Domingues C. M., Hunter J. R., Lambeck K. (2008). Understanding
global sea levels: past, present and future. Sustain. Sci..

[111] Fox-Kemper, B. , Hewitt, H. T. , Xiao, C. , A∂̷algeirsdóttir, G. , Drijfhout, S. S. , Edwards, T. L. , Golledge, N. R. , Hemer, M. , Kopp, R. E. , Krinner ; Ocean, cryosphere, and sea-level change. In Masson-Delmotte, V. , Zhai, P. ; Pirani, A. ; Connors, S. L. ; Péan, C. , , Eds.; Climate Change 2021: The Physical Science Basis. Contribution of Working Group I to the Sixth Assessment Report of the Intergovernmental Panel on Climate Change. 2021. (pp 1211–1362). Cambridge University Press. https://www.ipcc.ch/report/ar6/wg1.

[112] Landerer F. W., Jungclaus J. H., Marotzke J. (2007). Regional dynamic and
steric sea level change in response to the IPCC-A1B scenario. J. Phys. Oceanogr..

[113] Ross A. C., Najjar R. G., Li M., Mann M. E., Ford S. E., Katz B. (2015). Influences on decadal-scale variations
of salinity in a coastal plain estuary. Estua.,
Coast. Shelf Sci..

[114] Chua V. P., Xu M. (2014). Impacts of sea-level rise on estuarine
circulation: An idealized estuary and San Francisco Bay. J. Mar. Sys..

[115] Rice K. C., Hong B., Shen J. (2012). Assessment of salinity
intrusion in the James and Chickahominy Rivers as a result of simulated
sea-level rise in Chesapeake Bay, East Coast, USA. J. Environ. Manag..

[116] Lee J., Biemond B., de Swart H., Dijkstra H. A. (2024). Increasing
risks
of extreme salt intrusion events across European estuaries in a warming
climate. Commun. Earth Environ..

[117] Hong B., Liu Z., Shen J., Wu H., Gong W., Xu H., Wang D. (2020). Potential physical
impacts of sea-level rise on the Pearl River Estuary, China. J. Mar. Sys..

[118] Eslami S., Hoekstra P., Trung N. N., Kantoush S. A., Doan W. B., Do D. D., Qung T. T., van der
Vegt M. (2019). Tidal amplification and salt intrusion in the Mekong Delta driven
by anthropogenic sediment starvation. Sci. Rep..

[119] Lee J., Biemond B., van Keulen D., Huismans Y., van Westen R. M., de Swart H. E., Dijkstra H. A., Kranenburg W. M. (2025). Global
increases of salt intrusion in estuaries under future environmental
conditions. Nat. Commun..

[120] MacCready P., Geyer W. R. (2010). Advances in estuarine physics. Annu. Rev. Marine. Sci..

[121] Ross A. C., Najjar R. G., Li M., Lee S. B., Zhang F., Liu W. (2017). Fingerprints of sea-level
rise on
changing tides in the Chesapeake and Delaware Bays. J. Geophys. Res. Oceans.

[122] Burchard H., Schuttelaars H. M., Ralston D. K. (2018). Sediment Trapping
in Estuaries. Annu. Rev. Mar. Sci..

[123] Friedrichs, C. T. ; Armbrust, B. A. ; deSwart, H. E. Hydrodynamics and equilibrium sediment dynamics of shallow, funnel-shaped tidal estuaries; Balkema Press: 1998; https://scholarworks.wm.edu/handle/internal/19273.

[124] Meade R. H. (1969). Landward transport of bottom sediments
in estuaries
of the Atlantic coastal plain. J. Sediment.
Res..

[125] Klingbeil A. D., Sommerfield C. K. (2005). Latest Holocene evolution and human
disturbance of a channel segment in the Hudson River Estuary. Mar. Geol..

[126] Sallenger A. H., Doran K. S., Howd P. A. (2012). Hotspot
of accelerated sea-level rise on the Atlantic coast of North America. Nat. Clim. Change..

[127] Ezer T., Atkinson L. P., Corlett W. B., Blanco J. L. (2013). Gulf Stream’s
induced sea level rise and variability along the US mid-Atlantic coast. J. Geophys. Res. Oceans.

[128] Domingues R., Goni G., Baringer M., Volkov D. (2018). What caused
the accelerated sea level changes along the U.S. East Coast during
2010–2015?. Geophys. Res. Lett..

[129] Dangendorf S., Hendricks N., Sun Q., Klinck J., Ezer T., Frederikse T., Calfat F. M., Wahl T., Tornqvist T. E. (2023). Acceleration
of U.S. Southeast and Gulf coast sea-level
rise amplified by internal climate variability. Nat. Commun..

[130] Muis S., Haigh I. D., Guimarães
Nobre G., Aerts J. C. J. H., Ward P. J. (2018). Influence of El
Niño-Southern
Oscillation on global coastal flooding. Earth’s
Futur..

[131] Hamlington B. D., Leben R. R., Kim K.-Y., Nerem R. S., Atkinson L. P., Thompson P. R. (2015). The effect of the
El Nino-Southern
Oscillation on ∼ U.S. regional and coastal sea level. J. Geophys. Res. Oceans.

[132] Spillane M. C., Enfield D. B., Allen J. S. (1987). Intraseasonal oscillations
in sea level along the West Coast of the Americas. J. Phys. Oceanogr..

[133] Gong W., Lin Z., Zhang H., Lin H. (2022). The response
of salt intrusion to changes in river discharge, tidal range, and
winds, based on wavelet analysis in the Modaomen estuary, China. Ocean Coast. Manag..

[134] Polade S. D., Pierce D. W., Cayan D. R., Gershunov A., Dettinger M. D. (2014). The key role of dry days in changing
regional climate
and precipitation regimes. Sci. Rep..

[135] Douville, H. , Raghavan, K. , Renwick, J. , Allan, R. P. , Arias, P. A. , Barlow, M. , Cerezo-Mota, R. , Cherchi, A. ; Gan, T.Y. ; Gergis, J. ; Jiang, D. ; Khan, A. ; Pokam Mba, W. ; Rosenfeld, D. ; Tierney, J. ; Zolina, O. , 2021: Water Cycle Changes. In Climate Change 2021: The Physical Science Basis. Contribution of Working Group I to the Sixth Assessment Report of the Intergovernmental Panel on Climate Change [Masson-Delmotte, V., P. Zhai , Pirani, A. ; Connors, S.L. ; Péan, C. ; Berger, S. ; Caud, N. ; Chen, Y. ; Goldfarb, L. ; Gomis, M.I. ; Huang, M. ; Leitzell, K. ; Lonnoy, E. ; Matthews, J. B. R. ; Maycock, T. K. ; Waterfield, T. ; Yelekçi, O. ; Yu, R. ; Zhou, B. , Eds.; Cambridge University Press, Cambridge, United Kingdom and New York, NY, USA, pp 1055–1210, doi: 10.1017/9781009157896.010.

[136] Naumann G., Alfieri L., Wyser K., Mentaschi L., Betts R. A., Carrao H., Spinoni J., Vogt J., Feyen L. (2018). Global changes in drought conditions
under different levels of warming. Geophys.
Res. Lett..

[137] Pokhrel Y., Felfelani F., Satoh Y. (2021). Global
terrestrial water storage and drought severity under climate change. Nat. Clim. Chang..

[138] Cook B. I., Mankin J. S., Marvel K., Williams A. P., Smerdon J. E., Anchukaitis K. J. (2020). Twenty-first century drought projections
in the CMIP6 forcing scenarios. Earth’s
Futur..

[139] Li M., Zhong L., Boicourt W. C., Zhang S., Zhang D. (2007). Hurricane-induced
destratification and destratification in a partially-mixed estuary. J. Mar. Res..

[140] Feng J., Li D., Wang T., Liu Q., Deng L., Zhao L. (2019). Acceleration of the extreme sea level
rise along the Chinese coast. ESS.

[141] Chand S. S., Walsh K. J. E., Camargo S. J., Kossin J. P., Tory K. J., Wehner M. F., Chan J. C. L., Klotzbach P. J., Dowdy A. J., Bell S. S., Ramsay H. A., Murakami H. (2022). Declining
tropical cyclone frequency under global warming. Nat. Clim. Change.

[142] Camargo S. J., Murakami H., Bloemendaal N., Chand S. S., Deshpande M. S., Dominguez-Sarmiento C., González-Alemán J. J., Knutson T. R., Lin I. I., Moon I.-J., Patricola C. M., Reed K. A., Roberts M. J., Scoccimarro E., Tam C. Y., Wallace E. J., Wu L., Yamada Y., Zhang W., Zhao H. (2023). An update on the influence
of natural climate variability and anthropogenic climate change on
tropical cyclones. Trop. Cyclone Res. Rev..

[143] Klotzbach P. J., Wood K. M., Schreck C. J., Bowen S. G., Patricola C. M., Bell M. M. (2022). Trends in global
tropical cyclone activity: 1990–2021. Geophys. Res. Lett..

[144] Kossin J. P., Knapp K. R., Olander T. L., Velden C. S. (2020). Global
increase in major tropical cyclone exceedance probability over the
past four decades. Proc. Natl. Acad. Sci. U.S.A..

[145] Knutson T. R., Camargo S. J., Chan J. C. L. (2019). Tropical cyclones and
climate change assessment: Part I. Detection
and attribution. Bull. Am. Meteor. Soc..

[146] Wang S., Toumi R. (2021). Recent migration of tropical cyclones
toward coasts. Science.

[147] Pawlowicz R. (2015). The Absolute Salinity of seawater
diluted by riverwater. Deep-Sea Res. PART I.

[148] Pawlowicz R. (2008). Calculating the conductivity of natural
waters. L&O Methods.

[149] Raymond P. A., Oh N. H., Turner R. E., Broussard W. (2008). Anthropogenically
enhanced fluxes of water and carbon from the Mississippi River. Nature.

[150] Najjar R. G., Herrmann M., Cintron Del Valle S. M., Fredmann J. R., Friedrichs M. A. M., Harris L. A., Shadwick E. H., Stets E. G., Woodland R. J. (2020). Alkalinity in tidal tributaries of
the Chesapeake Bay. J. Geophys. Res. Oceans.

[151] Wahl T., Jain S., Bender J., Meyers S. D., Luther M. E. (2015). Increasing risk of compound flooding from storm surge
and rainfall for major US cities. Nat. Clim.
Change.

[152] Ye Y., Zhang J., Yu H., Sun W., Moghimi S., Myers E., Nunez K., Zhang R., Wang H. V., Roland A., Martins K., Bertin X., Du J., Liu Z. (2020). Simulating storm surge
and compound flooding events with a creek-to-ocean
model: Importance of baroclinic effects. Ocean
Modelling.

[153] Shen C., Testa J. M., Li M., Cai W.-J., Waldbusser G. G., Ni W., Kemp W. M., Cornwall J., Chen B., Brodeur J., Su J. (2019). Controls on carbonate
system dynamics in a coastal plain estuary: a modelling study. J. Geophysi. Res.-Biogeosci..

[154] Shen C., Testa J. M., Ni W., Cai W.-J., Li M., Kemp W. M. (2019). Ecosystem metabolism and carbon balance in Chesapeake
Bay: A 30-year analysis using a coupled hydrodynamic-biogeochemical
model. J. Geophys. Res.-Oceans.

[155] Li M., Li R., Cai W.-J., Testa J. M., Shen C. (2020). Effects of
wind-driven lateral upwelling on estuarine carbonate chemistry. Front. Mar. Sci..

[156] Li M., Guo Y., Cai W.-J., Testa J. M., Shen C., Li R., Su J. (2023). Projected increase in carbon dioxide drawdown and acidification
in large estuaries under climate change. Commun.
Earth Environ..

[157] Li M., Li R., Guo Y., Testa J. M., Cai W.-J., Shen C., Chen Y., Kaushal S. S. (2025). 2025. Disentangling
the effects of global and regional drivers on diverse long-term pH
trends in coastal waters. AGU Advances.

[158] Pawlowicz R., Feistel R. (2012). Limnological applications
of the
Thermodynamic Equation of Seawater 2010 (TEOS-10). L&O Methods.

[159] Lassiter A. (2024). Planning for drinking water salinization in the US
Atlantic and Gulf Coast regions. JAPA.

[160] Hansen K., Mullin M. (2022). Barriers to water infrastructure
investment: Findings from a survey of US local elected officials. PLoS Water.

[161] Quon H., Jiang S. (2023). Decision making for
implementing
non-traditional water sources: a review of challenges and potential
solutions. npj Clean Water.

[162] Delaware River Basin Commission (DRBC) , 2024. Flow Management. https://www.nj.gov/drbc/programs/flow/flow-mgmt.html. (accessed 2024-11-15).

[163] Lai V., Huang Y. F., Koo C. H., Ahmed A. N., El-Shafie A. (2022). A review of
reservoir operation optimizations: from traditional models to metaheuristic
algorithms. Arch. Computat. Methods Eng..

[164] Wilby R. L., Dessai S. (2010). Robust adaptation to
climate change. Weather.

[165] Giuliani M., Castelletti A., Pianosi F., Mason E., Reed P. (2016). Curses, tradeoffs,
and scalable management: advancing evolutionary
multiobjective direct policy search to improve water reservoir operations. J. Water Resour. Plan. Manag..

[166] Herman J. D., Quinn J. D., Steinschneider S., Giuliani M., Fletcher S. (2020). Climate adaptation as a control problem:
Review and perspectives on dynamic water resources planning under
uncertainty. Water Resour. Res..

[167] Giuliani M., Herman J. D., Castelletti A., Reed P. (2014). Many-objective reservoir policy identification and refinement to
reduce policy inertia and myopia in water management. Water Resour. Res..

[168] Giuliani M., Quinn J. D., Herman J. D., Castelletti A., Reed P. M. (2018). Scalable multiobjective control for large-scale water
resources systems under uncertainty. IEEE Trans.
Control Syst. Technol..

[169] Zaniolo M., Giuliani M., Castelletti A. (2021). Policy representation
learning for multiobjective reservoir policy design with different
objective dynamics. Water Resour. Res..

[170] Chen W., Olden J. D. (2017). Designing flows to resolve human
and environmental water needs in a dam-regulated river. Nat. Commun..

