# Supplementary material for: The Emerging Global Threat of Salt Contamination of Water Supplies in Tidal Rivers
Source: Environ Sci Technol Lett. 2025 Jul 2;12(8):881–92. doi: 10.1021/acs.estlett.5c00505 (PMC12351528; doi:10.1021/acs.estlett.5c00505)
Supplement: Supplementary file 1 [file ez5c00505_si_001.pdf]

Supporting Information

The emerging global threat of salt contamination of water supplies in tidal rivers

Ming Li[1*], Raymond Najjar[2], Sujay Kaushal[3], Alfonso Mejia[4], Robert Chant[5], David K. Ralston[6], Hans Burchard[7], Antonia Hadjimichael[8,9], Allison Lassiter[10], Xiaohong Wang[11]

[1]Horn Point Laboratory, University of Maryland Center for Environmental Science, USA

[2]Department of Meteorology and Atmospheric Science, The Pennsylvania State University, USA

[3]Department of Geology, University of Maryland, USA

[4]Department of Civil and Environmental Engineering, The Pennsylvania State University, USA

[5]Department of Marine and Coastal Sciences, Rutgers University, USA

[6]Applied Ocean Physics and Engineering Department, Woods Hole Oceanographic Institution, USA

[7]Department of Physical Oceanography, Leibniz Institute for Baltic Sea Research, Warnemünde, Germany

[8]Department of Geosciences, The Pennsylvania State University, University Park, PA, USA

[9]Earth and Environmental Systems Institute (EESI), The Pennsylvania State University, University Park, PA, USA

[10]Department of City and Regional Planning, Weitzman School of Design, University of Pennsylvania, USA

[11]Department of Computer Science, Salisbury University, USA

[*]Corresponding author: Ming Li (mingli@umces.edu)

This file contains three figures:

Figure S1

Figure S2

Figure S3

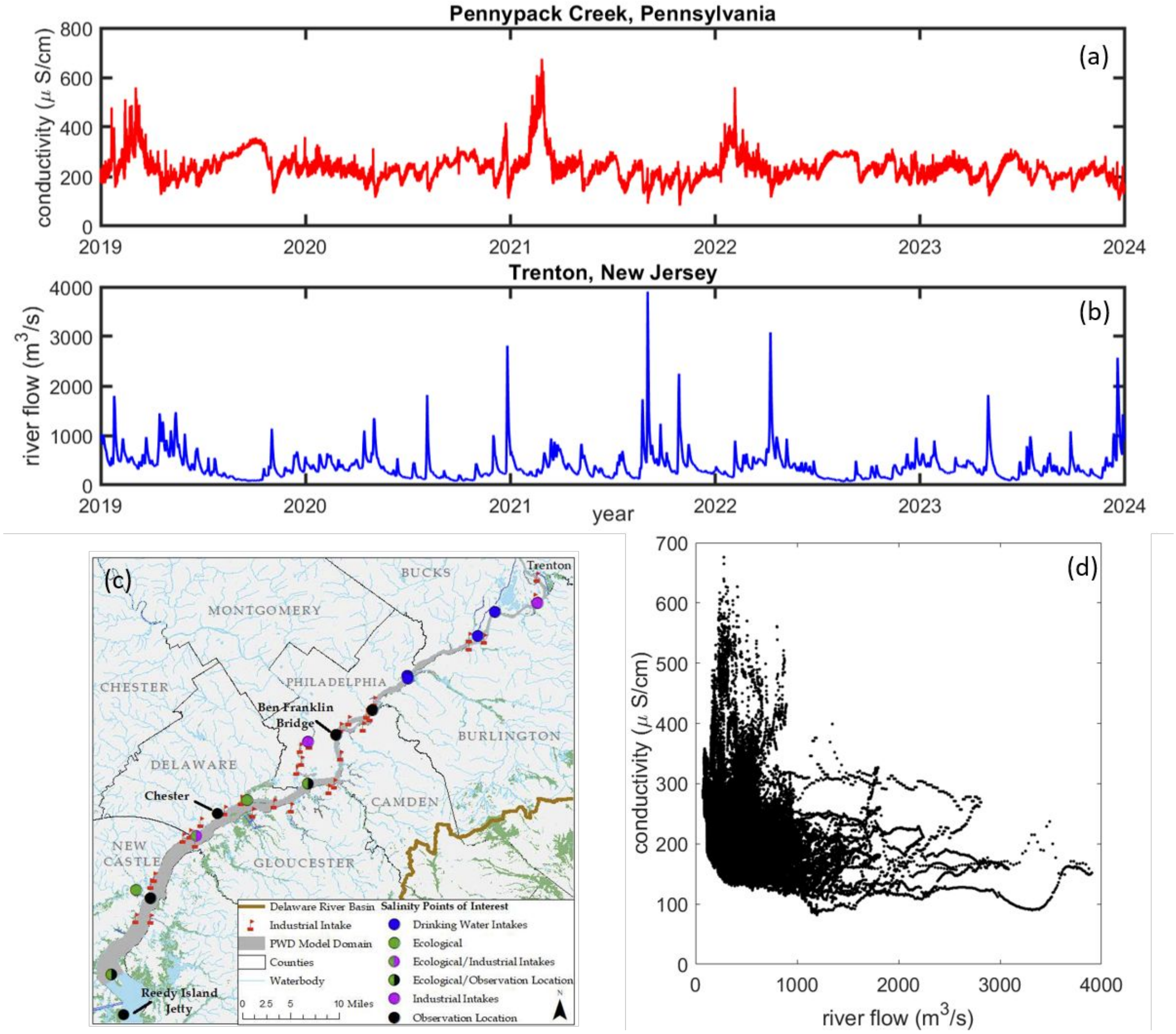

Figure S1. Time series of conductivity (a) at the US Geological Station (USGS) station at Pennypack Creek, Pennsylvania (near the water intakes for the City of Philadelphia) and the river flow (b) at the upstream station in Trenton, New Jersey during 2019–2024. Conductivity measures the ability of water to conduct electricity, which is directly related to the presence of dissolved ions (salts). Conductivity can be converted to salinity in oceanic waters but the relationship between conductivity and salinity is more complicated in tidal rivers due to varying composition of major salt ions. Three spikes of high conductivity (salinity) were observed during the winters of 2019, 2021 and 2022. (c) A map of the Delaware River and water intake locations (courtesy of Philadelphia Water Department). (d) A scatter plot of conductivity measurement at Pennypack versus the river flow at Trenton, showing an inverse relationship.

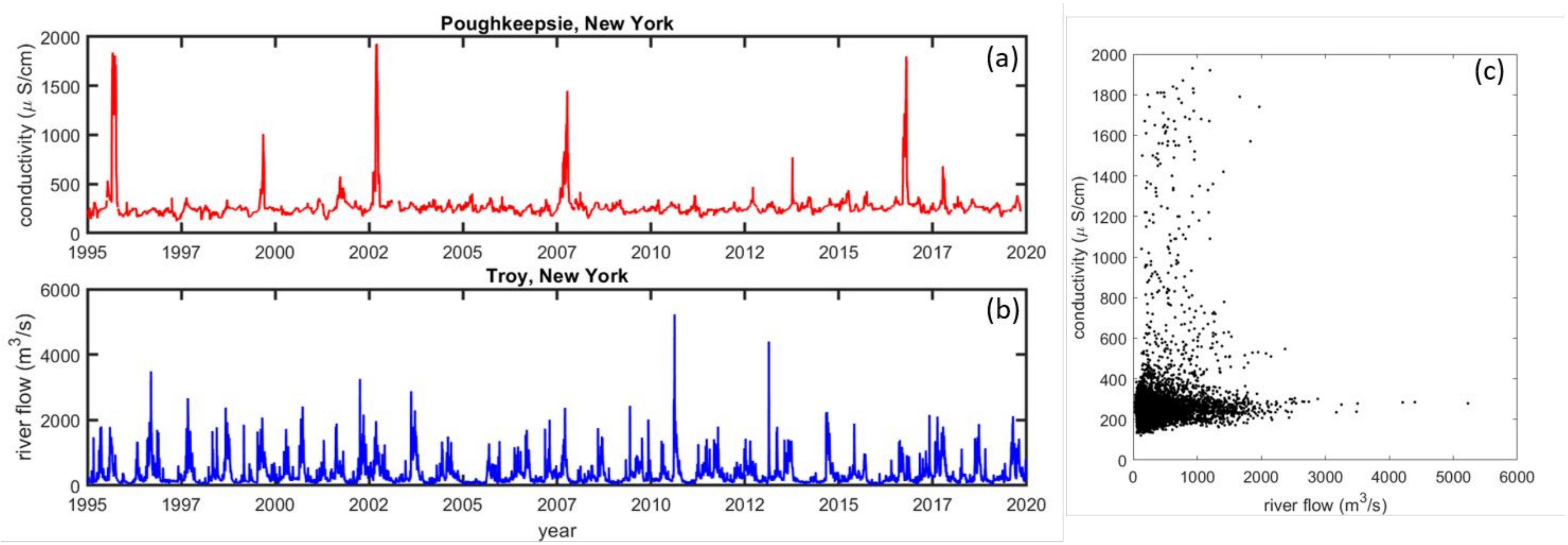

Figure S2. Time series of conductivity (a) at the USGS station at Poughkeepsie, New York and the river flow (b) at Troy, New York where the Upper Hudson and Mohawk Rivers converge and flow into the tidal Hudson River during 1995–2019. A number of high salinity spikes were observed during low-flow periods ($< \sim 200$ m^3 s^{-1}) in early fall. (c) A scatter plot of conductivity measurement at the USGS station at Poughkeepsie versus the river flow at Troy, showing that all high conductivity/salinity events occurred during low river flows.

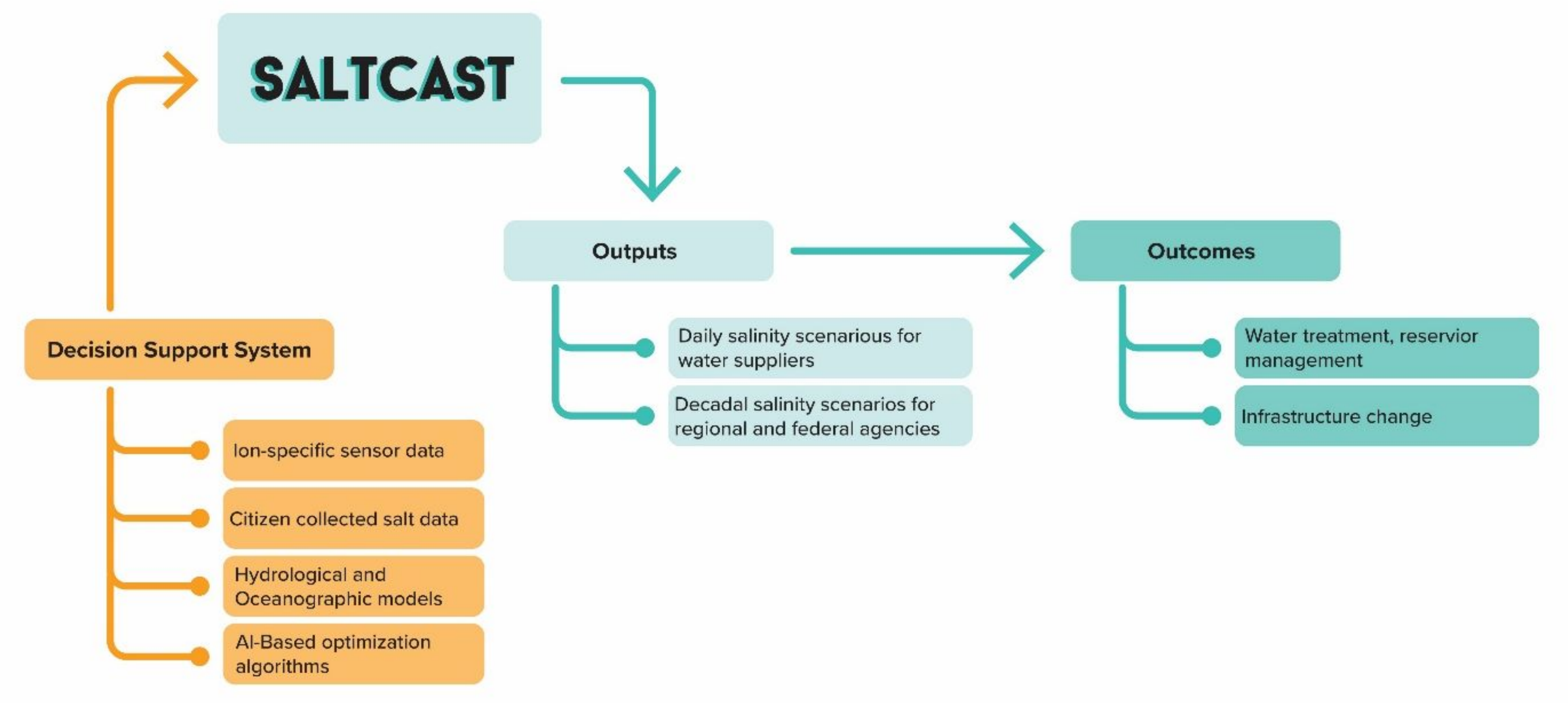

Figure S3. A schematic diagram illustrating various components of a decision support system (*SaltCast*) for salinity management in tidal rivers. A preliminary prototype of *SaltCast* can be found at saltcast.io. We propose the development of a web-based salinity management tool to ensure drinking water safety, sustainable freshwater uses and infrastructure integrity. *SaltCast* integrates advanced hydrological and oceanographic models, AI-based algorithms, and ion-specific sensors to deliver general and customizable information to support decision-making by communities, water resource managers, and industry users.
